# Efficacy of immune checkpoint inhibitors differs in various status of carcinoma: a study based on 29 cohorts with 3255 participants

**DOI:** 10.1007/s00262-024-03663-z

**Published:** 2024-03-30

**Authors:** Chunlan Wu, Yujun Ke, Luying Wan, Xianhe Xie

**Affiliations:** 1 Department of Oncology, Molecular Oncology Research Institute, the First Affiliated Hospital, Fujian Medical University, Fuzhou, 350005 China; 2 Department of Oncology, National Regional Medical Center, Binhai Campus of the First Affiliated Hospital, Fujian Medical University, Fuzhou, 350212 China; 3 Department of Anesthesiology, the First Affiliated Hospital, Fujian Medical University, Fuzhou, 350005 China; 4 Department of Anesthesiology, National Regional Medical Center, Binhai Campus of the First Affiliated Hospital, Fujian Medical University, Fuzhou, 350212 China; 5 Fujian Key Laboratory of Precision Medicine for Cancer, The First Affiliated Hospital of Fujian Medical University, Fuzhou, 350005 China

**Keywords:** Immune checkpoint inhibitors (ICIs), Viral infection, Malignancy, efficacy, Hot tumor microenvironment

## Abstract

**Background:**

Pre-clinical data have revealed that viral infection, such as Hepatitis B virus (HBV), Hepatitis C virus (HCV), and Human Papilloma virus (HPV), may lead to the development of “hot” or “immune-sensitive” tumors, which may impact the efficacy of immune checkpoint inhibitor (ICIs). Therefore, This study aimed to investigate the impact of viral status on the efficacy of ICIs.

**Methods:**

Electronic databases were searched to identify relevant trials. The primary endpoints were overall survival (OS) and progression-free survival (PFS) measured by hazard ratio (HR). Stratified analyses were accomplished based on viral types, treatment regimens, and patient locations.

**Results:**

A total of 3255 participants were recruited, including 252 cases of gastric cancer, 156 cases of nasopharyngeal carcinoma, 1603 cases of hepatocellular carcinoma, and 1244 cases of head and neck squamous cell carcinoma. Pooled results demonstrated a significant association between viral infection and favorable outcomes in patients receiving ICIs, including improved OS [HR = 0.67, 95%CI (0.57–0.79), *P* < 0.0001], increased ORR [OR = 1.43, 95%CI (1.14–1.80), *P* = 0.0018], and a trend toward enhanced PFS [HR = 0.75, 95%CI (0.56–1.00), *P* = 0.05]. In subgroup analyses, patients treated with ICIs who were exposed to HBV/HCV or HPV infection exhibited an evidently superior OS without heterogeneity, compared to those without infection.

**Conclusions:**

This study indicated that the presence of viral infection was evidently associated with improved outcomes in cancer patients undergoing ICIs, particularly in cases of HBV/HCV and HPV infections.

**Supplementary Information:**

The online version contains supplementary material available at 10.1007/s00262-024-03663-z.

## Introduction

Immune checkpoint inhibitors (ICIs) have revolutionized the field of cancer treatment [1–4]. This groundbreaking approach has shifted the focus from indiscriminate targeting of cancer cells by traditional chemotherapy to enhancing the immune system's ability to selectively attack tumor cells. However, the efficacy of ICIs is still far from satisfactory due to resistance [5, 6], and the distinction of biomarkers of response is an intense area of research [7]. This could potentially be linked to the variability observed in the tumor microenvironment (TME) across different types of tumors [8, 9]. Thus, it is crucial to identify the populations that benefit from ICIs treatment.

Based on the infiltration of T cells, Chen and Mellman have classified the tumor immune microenvironment into three different phenotypes: immune-desert, immune-excluded, and immune-inflamed. Among them, the immune-desert and immune-excluded phenotypes, also known as “cold tumors”, were non-inflamed tumors that were typically insensitive to ICIs. In contrast, the immune-inflamed phenotype, referred to as "hot tumors", exhibited a significantly stronger response to ICIs [8, 10]. Studies demonstrated that tumors associated viral infection often exhibit an “hot tumor” [11, 12]. However, the impact of viral infection in tumors on the efficacy of ICIs remains a topic of debate in clinical practice, with no established consensus. While some researchers supported a positive impact [13, 14], others advocated for non-inferior survival outcome [15, 16]. Additionally, the types of tumors, viruses and ICIs were various. Therefore, it remains to be fully illuminated that the impact of viral status on the efficacy of ICIs in cancer patients.

Previous studies had preliminarily explored the effect of human papilloma virus (HPV) on ICIs through meta-analysis [17], but they focused on single type of tumor and lack of subgroup analyses. Here, we conducted a comprehensive survey based on a large sample size (29 cohorts incorporating 3,255 individuals), multiple types of viruses and tumors to evaluate the impact of viral status on ICIs efficacy for malignancies.

## Materials and methods

### Literature searches

PubMed, Cochrane Library, and EMBASE were systematically searched to identify relevant studies up until December 15th, 2023 by entering the following keywords: “immune checkpoint inhibitors”, “ICI”, “immunotherapy”, “PD-1”, “PD-L1”, “CTLA-4”, “programmed cell death protein 1”, “programmed cell death protein ligand 1”, “cytotoxic T lymphocyte-associated protein 4”, “pembrolizumab”, “nivolumab”, “atezolizumab”, “ipilimumab”, “tremelimumab”, “avelumab”, “durvalumab”, “carelizumab”, “tislelizumab”, “cemiplimab”, “toripalimab”, “penpulimab”, “cemiplimab”, “adebrelimab”, “sugemalimab”, “Epstein Barr virus”, “EBV”, “Hepatitis B virus”, “HBV”, “Human Papilloma virus”, “HPV”, “hepatitis C virus”, “HCV”, “gastric cancer”, “GC”, “stomach Cancer”, “hepatic cancer”, “liver cancer”, “HCC”, “nasopharyngeal carcinoma”, “NPC”, “head and neck cancer”, “HNSCC”, “lymphoma”, “cancer”, “neoplasm”, “tumor”, “carcinoma”, and “malignancy”. Moreover, the reference lists of related articles were scrutinized for additional studies.

### Selection of studies

Two investigators respectively performed an initial screening of titles and abstracts, and then scrutinized the full texts to identify eligible studies.

### Inclusion criteria

Inclusion criteria were included: (1) individuals were pathologically confirmed as malignancies; (2) therapeutic outcomes were analyzed on the efficacy of ICIs according to viral status (including Epstein Barr virus (EBV), hepatitis B virus (HBV), hepatitis C virus (HCV), and HPV; (3) A hazard ratio (HR) accompanied by a 95% confidence interval (CI) for progression-free survival (PFS) and/or overall survival (OS) and/or odds ratios (OR) with 95% CI for objective response rate (ORR) could be obtained or calculated from the original literature.

### Data extraction

Data from all enrolled studies were independently collected by two investigators. The data was collected from each publication as follow: publication year, first author, number of patients, primary tumor, immunotherapy agents, viral types, HR for OS and/or PFS, and OR for ORR between the viral infection group and viral uninfection group.

### Quality assessment

The quality of studies was assessed using the Newcastle–Ottawa quality assessment scale (NOS), with scores of more than six indicating medium to high quality [18]. Discrepancies were settled through a consensus reached among all investigators.

### Statistical methods

The primary endpoints of the study were OS and PFS. The association between viral status (infection vs uninfection) and the efficacy ICIs was measured applying HR with the corresponding 95% CI. Subgroup analyses were accomplished based on the viral types, treatment regimen, patient locations, and ICI agents. Statistical analysis was performed by R 4.2.2 statistical software. Heterogeneity was evaluated through the *I*-square tests and Cochran’s Q test. if *P* < 0.05 or *I*^2^ > 50%, it indicated remarkable heterogeneity, and a random effect model was employed. Otherwise, a fixed effect model was adopted. Publication bias was assessed using funnel plot, Egger’s test, and trim-and-fill method [19].

## Results

### Study selection and characteristics of trials

**Fig. 1:**
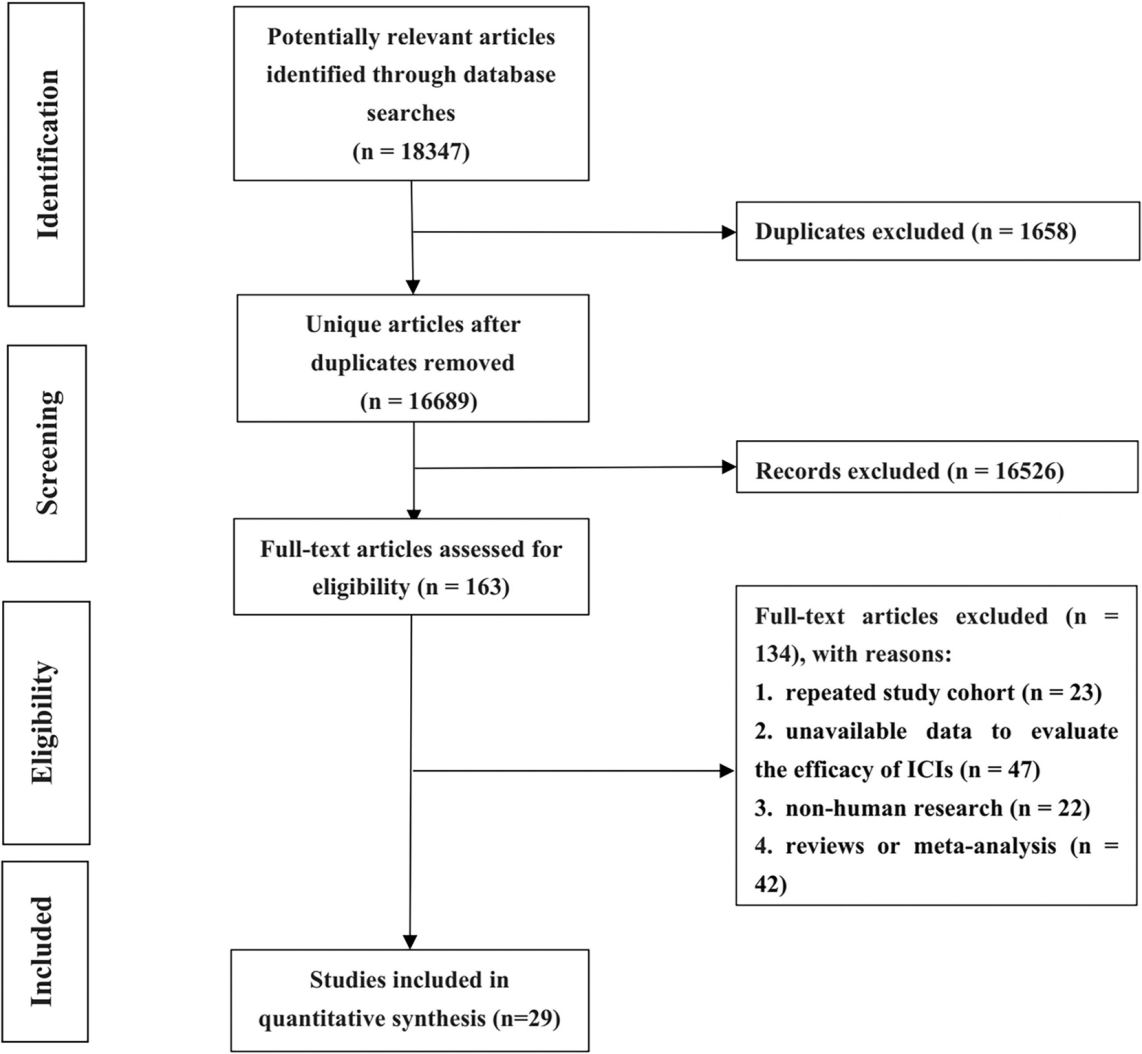
Flowchart on selection including trials in the meta-analysis

A total of 18,347 potentially relevant articles were intensively scrutinized. Among them, 1,658 were removed for duplication, while 16,689 were filtered out for digressing from the subject after screening the titles and abstracts. Subsequently, the full texts of 163 articles were thoroughly reviewed, of which 134 were excluded for the following reasons: repeated study cohort (n = 23), unavailable data to evaluate the efficacy of ICIs (n = 47), non-human research (n = 22), reviews or meta-analysis (n = 42). Finally, a total of 29 studies incorporating 3,255 participants were identified (The links of original article and details were shown in Supplementary 1). The elaborate procedure is displayed in Fig. 1.

A total of 3255 individuals in 13 retrospective studies and 16 prospective studies were recruited. All 29 adopted studies were rated as moderate or high quality. Furthermore, the sample size ranged from 12 to 421. Of these studies, 3 focused on gastric cancer (GC), 1 on nasopharyngeal carcinoma (NPC), 14 on hepatocellular carcinoma (HCC), 11 on headneck squamous cell carcinoma (HNSCC). Principal traits and details were presented in Table 1.

**Table 1:** The principal characteristics and further details of eligible articles

Author	Year	Patients location	Study type	Cancer type	Viral type	ICI agents	Number of patients		Male (%)
							Uninfection	Infection	
Chang [S1]	2022	China	R^c^	GC^e^	EBV^k^	Nivo^r^ or sintilimab or tislelizumab	107	19	74 (58)
Kim [S2]	2020	Korea	R	GC	EBV	Pembro^s^ or Nivo	56	4	37 (59)
Bai [S3]	2022	China	R	GC	EBV	ICIs^t^	44	22	49 (74)
Yang [S4]	2021	China	P^d^	NPC^f^	EBV	Camrelizumab	39	117	124 (80)
Liu [S5]	2022	China	R	HCC^g^	HBV^m^	Camrelizumab	26	28	43 (77)
Wu [S6]	2022	China	P	HCC	HBV or HCV^n^	Pembro	18	53	62 (87)
Yau [S7]	2019	Multi	P	HCC	HBV or HCV	Nivo	89	93	65 (76)
Wu [S8]	2022	China	R	HCC	HBV or HCV	Nivo	5	35	29 (73)
Yao [S9]	2021	China	R	HCC	HBV	ICIs	12	124	115 (85)
Sun [S10]	2022	China	R	HCC	HBV	ICIs	13	71	69 (82)
Kim [S11]	2021	Korea	R	HCC	HBV or HCV	Nivo	17	85	87 (85)
El-Khoueiry [S12]	2017	Multi^a^	P	HCC	HBV or HCV	Nivo	57	101	171 (80)
Ju [S13]	2022	China	P	HCC	HBV	Camrelizumab	15	65	66 (83)
Xin [S14]	2022	China	R	HCC	HBV	Atezo^u^	5	47	46 (89)
Zhu [S15]	2018	Multi	P	HCC	HBV or HCV	Pembro	81	22	86 (83)
Verset [S16]	2020	Multi	P	HCC	HBV or HCV	Pembro	29	20	44 (86)
Tomonari [S17]	2022	Japan	R	HCC	HBV or HCV	Atezo	33	38	58 (82)
Tada [S18]	2022	Japan	R	HCC	HBV or HCV	Atezo	208	213	340 (81)
Ferris [S19]	2021	USA and Europe^b^	P	HNSCC^h^	HPV^q^	Nivo	26	26	38 (73)
Powell [S20]	2020	USA	P	HNSCC	HPV	Pembro	25	34	50 (85)
Bauml [S21]	2017	Multi	P	HNSCC	HPV	Pembro	131	37	138 (81)
Chow [S22]	2016	Multi	P	HNSCC	HPV	Pembro	104	28	110 (83)
Black-Mono [S23]	2023	USA	R	HNSCC	HPV	Pembro	165	163	330 (77)
Black-combination [S23]	2023	USA	R	HNSCC	HPV	Pembro	93	70	170 (79)
Zandberg [S24]	2019	USA and Europe	P	HNSCC	HPV	Durva	65	34	NA^v^
Kim [S25]	2020	Korea	R	HNSCC	HPV	Pembro or Nivo	5	7	25 (71)
Leddon [S26]	2022	USA	P	HNSCC	HPV	Nivo	16	10	27 (69)
Seiwert [S27]	2018	USA and Israel	P	HNSCC	HPV	Pembro	37	23	49 (82)
Colevas [S28]	2018	USA	P	HNSCC	HPV	Atezo	12	13	27 (84)
Ferris [S29]	2018	Multi	P	HNSCC	HPV	Nivo	56	64	NA

Author	Median age	Combination drug	Line of therapy	PFS ^@^(months)		OS^#^ (months)		Quality evaluation
				Uninfection	Infection	Uninfection	Infection	
Chang [S1]	57 (37–78)	Mono^&^	2nd-line	3.5 (3.3–3.7)	3.8 (3.3–4.2)	NA	NA	7
Kim [S2]	54 (29–82)	Mono	2nd-line or late	NA*	NA	NA	NA	8
Bai [S3]	NA	CTLA-4i^$^ or Mono	1st-line or later	NA	NA	NA	NA	8
Yang [S4]	NA	Mono	3rd-line or late	6.0 (2.9–11.1)	2.7 (1.9–3.9)	22.7 (15.2-NR^β^)	16.5 (13.5–19.5)	9
Liu [S5]	Infection: 55.9 ± 11.5 Uninfection: 59.5 ± 10.9	Mono	1st-line or later	6.7 (5.0–8.4)	9.2 (7.4–11.0)	11.1 (9.7–12.5)	13.3 (11.4–15.2)	8
Wu [S6]	63 (28–89)	Lenvatinib	1st-line or later	NA	NA	NA	NA	8
Yau [S7]	63 (19–81)	Mono	2nd-line	NA	NA	15.1 (11.7–18.9)	HBV^К^ group 14.8 (9.1–20.2); HCV^α^ group 18.8 (11.2–30.8)	9
Wu [S8]	58.5 ± 13.8	Lenvatinib	1st-line or later	NA	NA	12.4	HBV group: NR HCV group: 23.2 (NR)	7
Yao [S9]	58 (14–84)	Antiangiogenic therapy	1st-line or later	NA	NA	NA	NA	7
Sun [S10]	53 (25–78)	Lenvatinib	1st-line or later	NA	NA	NA	NA	7
Kim [S11S1]	61 (54–69)	Mono	1st-line or later	NA	NA	NA	NA	7
El-Khoueiry [S12]	64 (56–70)	Mono	1st-line or later	4.0 (2.6–6.7)	4.0 (1.3–4.1)	13.2 (8.6-NE^w^)	NR^y^	8
Ju [S13]	52 (46–62)	Apatinib	1st-line or later	NA	NA	NA	NA	8
Xin [S14]		Beva^Ф^	NA	NA	NA	NA	NA	7
Zhu [S15]	68 (62–73)	Mono	2nd-line	NA	NA	NA	NA	8
Verset [S16]	68 (41–91)	Mono	1st-line	NA	NA	NA	NA	9
Tomonari[S17]	71 (66–79)	Beva	2nd-line or late	NA	NA	NA	NA	7
Tada [S18]	NA	Beva	2nd-line or late	NA	NA	NA	NA	7
Ferris [S19]	Infection: 63 (34–82) Uninfection: 60 (42–85)	Mono	1st-line	NA	NA	49.8 (12.4-NE)	NR	9
Powell [S20]	60 (36–81)	Cisplatin	1st-line	NA	NA	NA	NA	9
Bauml [S21]	61 (33–90)	Mono	2nd-line or late	NA	NA	NA	NA	9
Chow [S22]	60 (25–84)	Mono	1st-line or later	NA	NA	NA	NA	9
Black-Mono [S23]	69 (68–70)	Mono	1st-line	NA	NA	NA	NA	7
Black-Combination [S23]	64 (63–65)	Chemotherapy	1st-line	NA	NA	NA	NA	7
Zandberg [S24]	NA	Mono	2nd-line	NA	NA	5 (3.4–8.4)	10.2 (7.2–16.3)	9
Kim [S25]	58 (39–73)	Mono	1st-line or later	1.8 (1.3–2.3)	4.5 (0.0–11.0)	6.8 (1.1–12.6)	NR	8
Leddon [S26]	68 (49–85)	Mono	1st-line	NA	NA	NA	NA	9
Seiwert [S27]	63 (20–83)	Mono	1st-line or later	2 (2–4)	4 (2–10)	8 (4-NR)	NR (8-NR)	9
Colevas [S28]	62 (32–78)	Mono	1st-line or later	NA	NA	NA	NA	9
Ferris [S29]	59 (29–83)	Mono	2nd-line or late	NA	NA	7.7 (4.8–13.0)	9.1 (6.5–11.8)	9

^a^: Multi countries; ^b^:Europe; ^c^: Retrospectively; ^d^: Prospectively; ^e^: Gastric cancer; ^f^: Nasopharyngeal carcinoma; ^g^: Hepatocellular carcinoma; ^h^: Head and neck squamous cell carcinoma; ^k^: Epstein Barr virus; ^m^: Hepatitis B virus; ^n^: Hepatitis C virus; ^q^: Human Papilloma virus; ^r^: Nivolumab; ^s^: Pembrolizumab; ^t^: Immune checkpoint inhibitors; ^u^: Atezolizumab; ^v^: No answer; [S1–S29]: The links of original article and details were shown in Supplementary 1

^@^:Progression-free survival; ^#^: Overall survival; *: No answer; ^&^: Monotherapy; ^$^: Cytotoxic T lymphocyte-associated protein 4 inhibitor; ^β^: No rearch; ^К^: Hepatitis B virus; ^α^: Hepatitis C virus; ^Ф^: Bevacizumab; ^w^: Not estimable; [S1-S29]: The links of original article and details were shown in Supplementary 1

### Main results

#### The impact of viral status on malignancy patients treated with ICIs

**Fig. 2:**
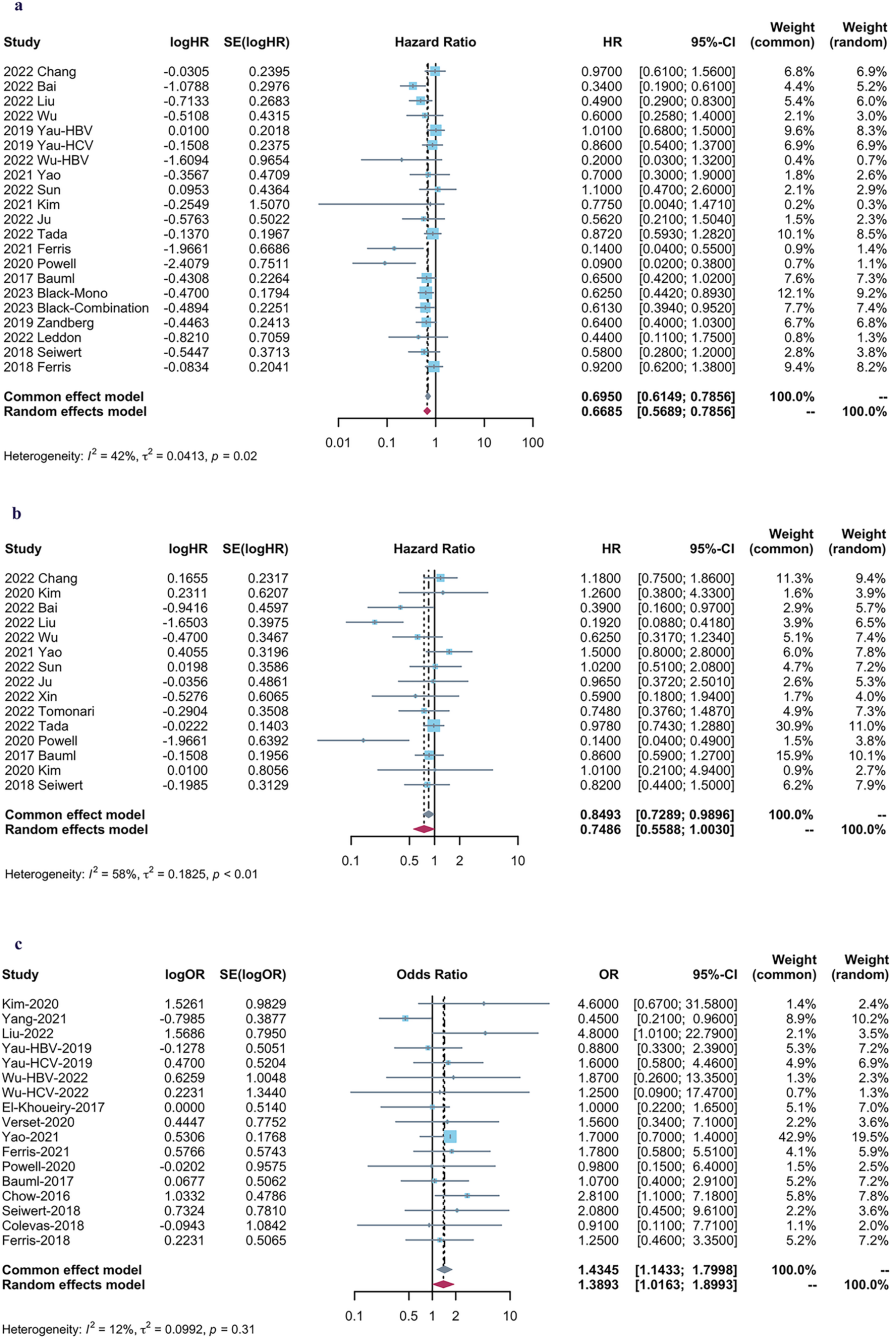
Forest plots for **a** overall survival (OS), **b** progression-free survival (PFS), and **c** objective response rate (ORR)

Pooled results showed that tumor patients with viral infection who received ICI agents had a significantly favorable OS [HR = 0.67, 95%CI (0.57–0.79), *P* < 0.0001] by a random-effect model (*I*^2^ = 42%, *P* = 0.02) (Fig. 2a), and a trend towards improved PFS [HR = 0.75, 95%CI (0.56–1.00), *P* = 0.05] based on a random-effect model (I2 = 58%, *P* < 0.01)(Fig. 2b). Furthermore, There was an increased ORR [OR = 1.43, 95%CI (1.14–1.80), *P* = 0.0018] in viral positive group according to a fixed-effect model (*I*2 = 12%, *P* = 0.31) (Fig. 2c).

#### Subgroup analysis for the impact of viral status

**Fig. 3:**
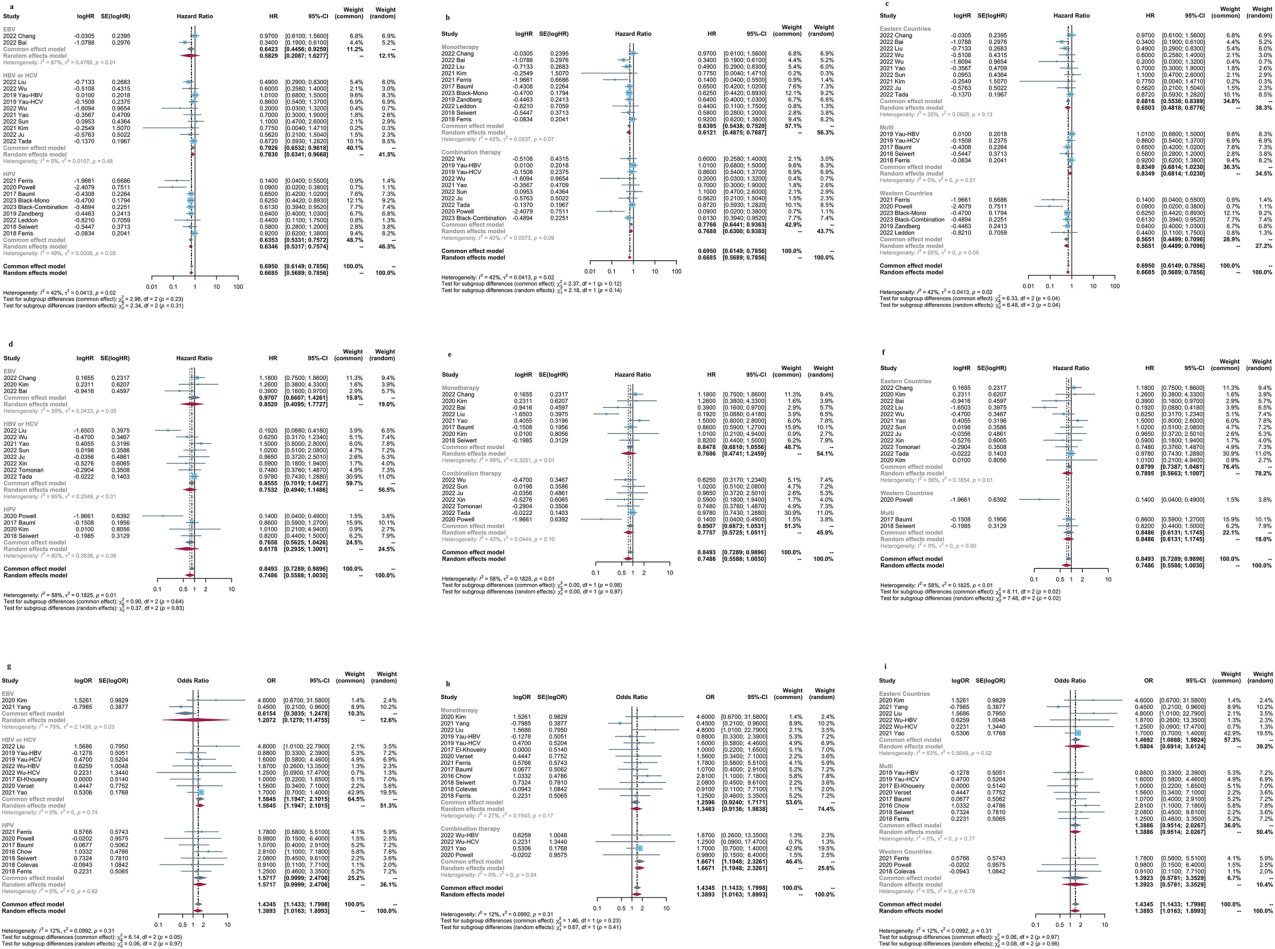
**a** The pooled HRs for overall survival (OS) stratified on viral types (EBV, HBV/HCV, and HPV); **b** treatment regions (monotherapy or combined therapy); **c** patients locations (western countries and eastern countries); **d** the pooled HRs for progression-free survival (PFS) stratified on viral types; **e** treatment regions; **f** patients locations; **g** the pooled ORs for objective response rate (ORR) stratified on viral types; **h** treatment regions; **i** patients locations

We performed subgroup based on the viral types, treatment regimen, and patient locations (Fig. 3a–i). The result showed that patients treated with ICIs who were exposed to HBV/HCV or HPV infection exhibited an evidently superior OS without heterogeneity, compared to those without HBV/HCV [HR = 0.79, 95%CI (0.65–0.96)] and HPV [HR = 0.64, 95%CI (0.53–0.76)]. While the OS was similar between the EBV-positive group and the EBV-negative group [HR = 0.58, 95%CI (0.21–1.63)] (Fig. 3a). Furthermore, we found that patients with viral infection who received ICIs had a significantly better OS, in contrast to those without infection, regardless of the treatment types (monotherapy or combined therapy) [HR = 0.64, 95%CI (0.54–0.75) and HR = 0.78, 95%CI (0.64–0.94), respectively] (Fig. 3b) and patient locations (eastern countries or western countries) [HR = 0.68, 95%CI (0.55–0.84) and HR = 0.57, 95%CI (0.45–0.71), respectively] (Fig. 3c). Additionally, the groups with HBV/HCV or HPV infection achieved a higher ORR compared to the groups without HBV/HCV or HPV infection)[OR = 1.58, 95%CI (1.19–2.10) and OR = 1.57, 95%CI (1.0–2.47), respectively] (Fig. 3g).

### Publication bias

**Fig. 4:**
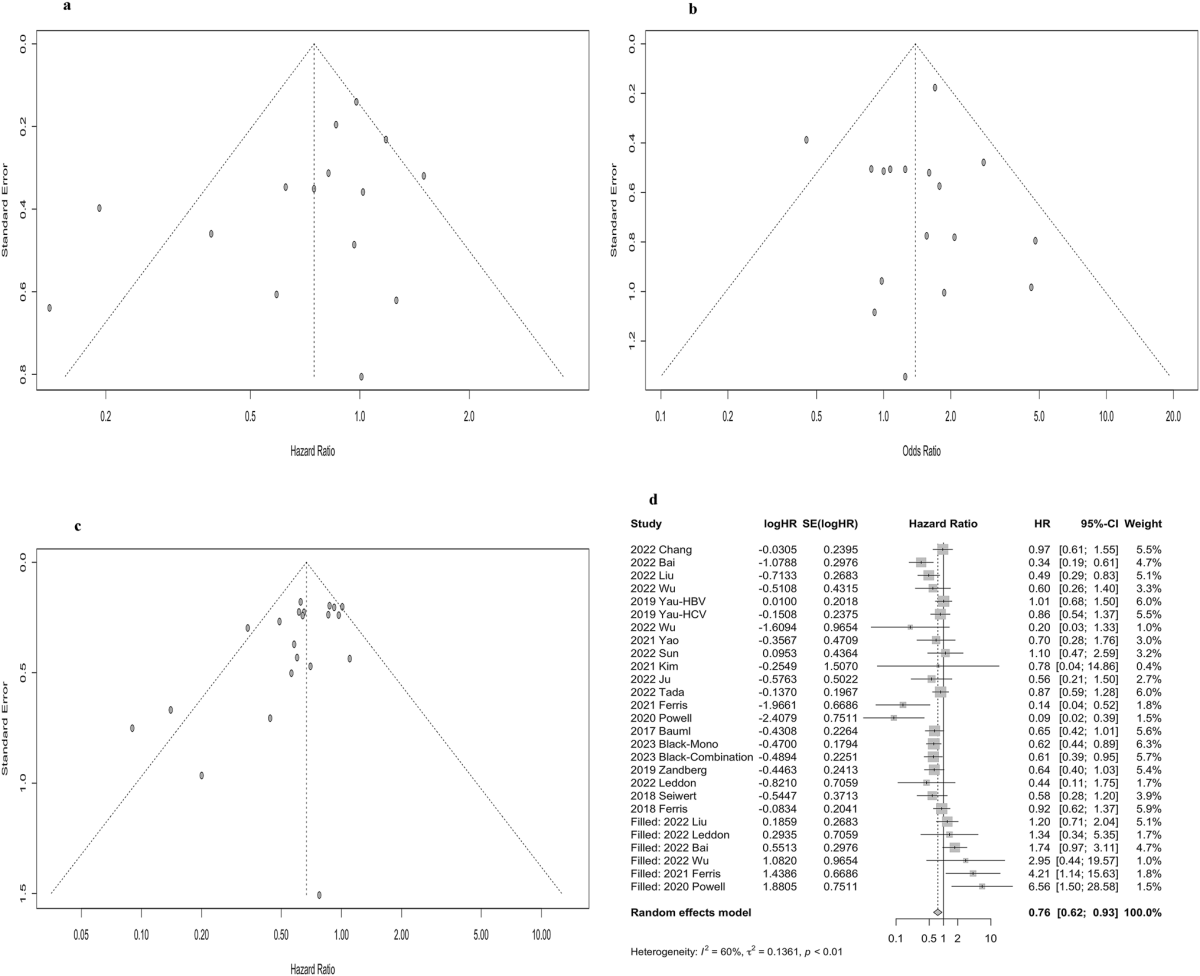
Funnel plot of publication bias on **a** progression-free survival (PFS), **b** objective response rate (ORR), and **c** overall survival (OS) in the meta-analysis; **d** the corrected HRs for OS based on the trim and fill method

The shape of the funnel plot suggested no publication bias for recruited studies on PFS (Egger: *P* = 0.11) (Fig. 4a) and ORR (Egger: *P* = 0.98) (Fig. 4b). However, there was a publication bias for OS (Egger: *P* = 0.02) (Fig. 4c). Nevertheless, in the results of the trim and fill method, the publication bias corrected overall effect size was 0.76 (95% CI: 0.62–0.93) (Fig. 4d), even though the effect size increased compared to the original ones. This implied that the results obtained for this study were reliable and consistent.

### The sensitivity analysis

**Fig. 5:**
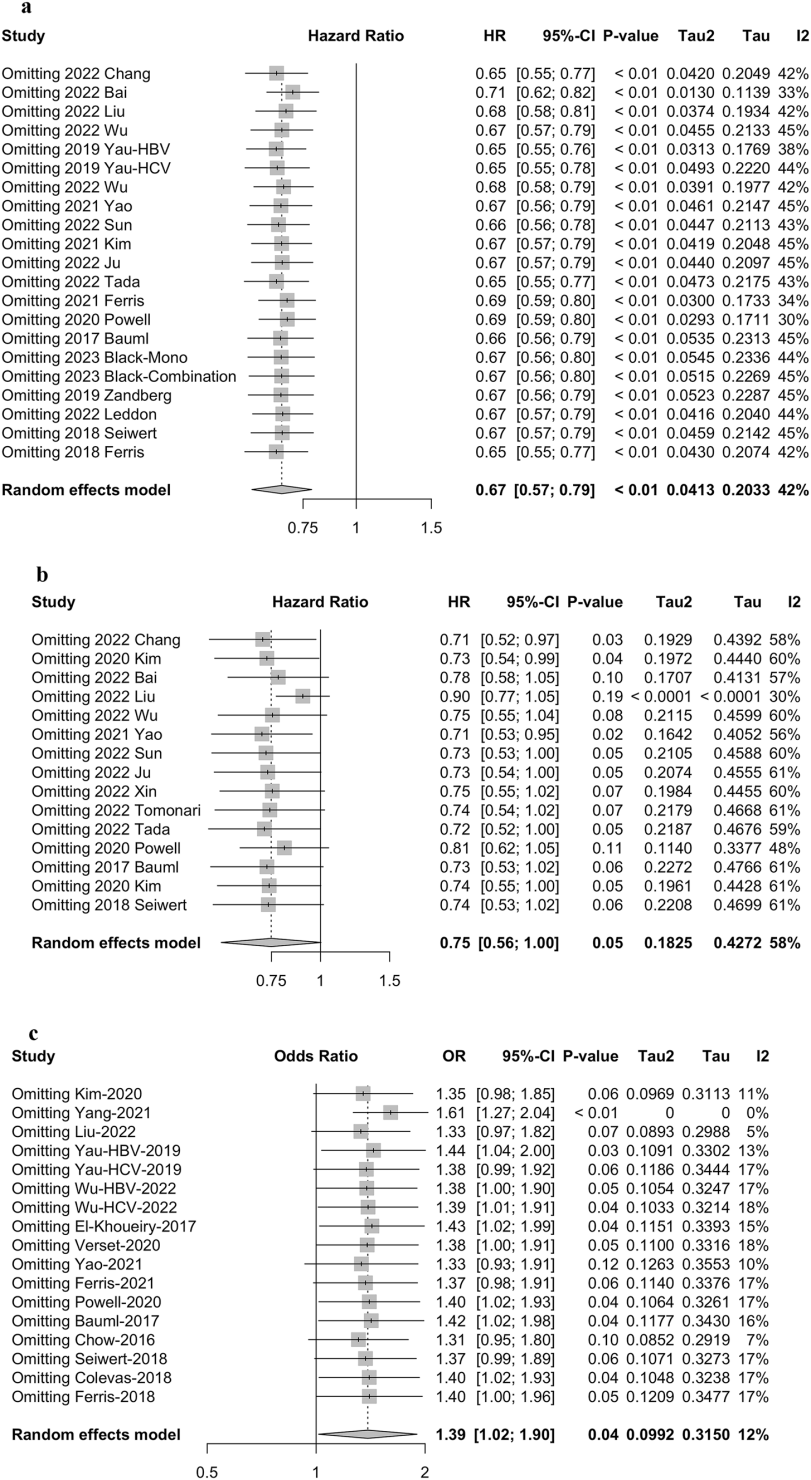
Sensitivity analysis of **a** overall survival (OS), **b** progression-free survival (PFS), and **c** objective response rate (ORR)

Sensitivity analyses were performed by excluding one single study from the primary analyses. The results showed that no single study significantly influenced the pooled HRs or ORs, suggesting that the data of this meta-analysis were relatively credible and stable (Fig. 5).

## Discussion

Recently, increasing evidences highlighted the importance of viral infection in influencing the efficacy of immunotherapy. Pre-clinical and clinical evidences have recognized that the viral infection may play a crucial role in boosting the immune response and improve prognosis of cancer patients undergoing ICIs treatment [11–14]. To the best of our knowledge, this study was firstly investigated the impact of viral infection on outcomes of cancer patients treated with ICIs based on a comprehensive survey (29 cohorts incorporating 3,255 individuals), multiple viruses types (EBV, HBV, HCV, and HPV) and multiple tumor types (including GC, NPC, HCC and HNSCC). The result demonstrated a significant association between viral infection and improved outcomes for cancer patients receiving ICIs treatment.

Mechanically, PD-1 and its ligands played a crucial role in enabling tumor cells to evade the anti-tumor response of immune system [20]. Less widely recognized was that the PD-1/PD-L1 axis also played a role in regulating immune responses against viral infection and can be influenced by various viruses [21, 22]. Upregulation of PD-1 and its ligands PD-L1 were observed during acute viral infection and after infection with persistent viruses including important human pathogens such as HBV, HCV, and EBV [23–26]. Moreover, viral infection associated carcinomas were typically characterized by abundant immune cell infiltration [11, 12], which might further positively affect the efficacy of ICIs.

Notably, our study exhibited that EBV infection did not impact the efficacy of ICI treatment, despite the frequent association of EBV infection with high PD-L1 expression in tumors was discovered [25, 26]. The reason remains to be elucidated. However, it should be noted this study comprising EBV associated tumors was only included 4 cohorts with a total of 408 participants. Therefore, caution should be advised when interpreting the result.

However, this study encountered two flaws: firstly, some of recruited studies were retrospective, although we had comprehensively analyzed the articles; secondly, due to the limited availability of comprehensive data, subgroup analysis based on specific ICI agents could not be conducted.

In conclusion, this study demonstrated that the presence of viral infection was positively associated with better outcomes, with improved OS, increased ORR, and potential benefits in PFS in cancer patients undergoing ICIs therapy. And subgroup analyses on therapy regimen and patient locations exhibited similar results, indicating the positive impact of viral infection on ICIs therapy in clinical practice.

### Supplementary Information

Below is the link to the electronic supplementary material.

Supplementary file1 (DOCX 16 KB)

## Data Availability

All data and material analyzed during this study are included in the published article.
