# Supplementary material for: Efficacy of immune checkpoint inhibitors differs in various status of carcinoma: a study based on 29 cohorts with 3255 participants
Source: Cancer Immunol Immunother. 2024 Mar 30;73(5):79. doi: 10.1007/s00262-024-03663-z (PMC10981616; doi:10.1007/s00262-024-03663-z)
Supplement: Supplementary file 1 — Supplementary file1 (DOCX 16 KB) [file 262_2024_3663_MOESM1_ESM.docx]

Supplementary 1

recruited studies [S1-S29]

S1. Chang C, Pei Y, Xu J, Zhang W, Zhang J, Shi S (2022) The full management from first-line to third-line treatments in patients with Her-2-negative advanced gastric cancer. Front Oncol 12: 949941. <https://doi.org/10.3389/fonc.2022.949941>

S2. Kim J, Kim B, Kang SY, Heo YJ, Park SH, Kim ST et al (2020) Tumor Mutational Burden Determined by Panel Sequencing Predicts Survival After Immunotherapy in Patients With Advanced Gastric Cancer. Front Oncol 10: 314. <https://doi.org/10.3389/fonc.2020.00314>

S3. Bai Y, Xie T, Wang Z, Tong S, Zhao X, Zhao F et al (2022) Efficacy and predictive biomarkers of immunotherapy in Epstein-Barr virus-associated gastric cancer. J Immunother Cancer 10(3): e004080. <https://doi.org/10.1136/jitc-2021-004080>

S4. Yang Y, Zhou T, Chen X, Li J, Pan J, He X et al (2021) Efficacy, safety, and biomarker analysis of Camrelizumab in Previously Treated Recurrent or Metastatic Nasopharyngeal Carcinoma (CAPTAIN study). J Immunother Cancer 9(12): e003790. <https://doi.org/10.1136/jitc-2021-003790>

S5. Liu H, Qin X, Xu Z, Wu M, Lu T, Zhou S et al (2022) Comparison of effectiveness and safety of camrelizumab between HBV-related and non-B, non-C hepatocellular carcinoma: A retrospective study in China. Front Genet 13: 1000448. <https://doi.org/10.3389/fgene.2022.1000448>

S6. Wu CJ, Lee PC, Hung YW, Lee CJ, Chi CT, Lee IC et al (2022) Lenvatinib plus pembrolizumab for systemic therapy-naïve and -experienced unresectable hepatocellular carcinoma. Cancer Immunol Immunother 71(11): 2631-2643. <https://doi.org/10.1007/s00262-022-03185-6>

S7. Yau T, Hsu C, Kim TY, Choo SP, Kang YK, Hou MM et al (2019) Nivolumab in advanced hepatocellular carcinoma: Sorafenib-experienced Asian cohort analysis. J Hepatol 71(3): 543-552. <https://doi.org/10.1016/j.jhep.2019.05.014>

S8. Wu WC, Lin TY, Chen MH, Hung YP, Liu CA, Lee RC et al (2022) Lenvatinib combined with nivolumab in advanced hepatocellular carcinoma-real-world experience. Invest New Drugs 40(4): 789-797. <https://doi.org/10.1007/s10637-022-01248-0>

S9. Yao J, Zhu X, Wu Z, Wei Q, Cai Y, Zheng Y et al (2022) Efficacy and safety of PD-1 inhibitor combined with antiangiogenic therapy for unresectable hepatocellular carcinoma: A multicenter retrospective study. Cancer Med 11(19): 3612-3622. <https://doi.org/10.1002/cam4.4747>

S10. Sun X, Zhang Q, Mei J, Yang Z, Chen M, Liang T (2022) Real-world efficiency of lenvatinib plus PD-1 blockades in advanced hepatocellular carcinoma: an exploration for expanded indications. BMC Cancer 22(1): 293. <https://doi.org/10.1186/s12885-022-09405-7>

S11. Kim N, Yu JI, Park HC, Yoo GS, Choi C, Hong JY et al (2021) Incorporating sarcopenia and inflammation with radiation therapy in patients with hepatocellular carcinoma treated with nivolumab. Cancer Immunol Immunother 70(6): 1593-1603. <https://doi.org/10.1007/s00262-020-02794-3>

S12. El-Khoueiry AB, Sangro B, Yau T, Crocenzi TS, Kudo M, Hsu C et al (2017) Nivolumab in patients with advanced hepatocellular carcinoma (CheckMate 040): an open-label, non-comparative, phase 1/2 dose escalation and expansion trial. Lancet 389(10088): 2492-2502. <https://doi.org/10.1016/s0140-6736(17)31046-2>

S13. Ju S, Zhou C, Hu J, Wang Y, Wang C, Liu J et al (2022) Late combination of transarterial chemoembolization with apatinib and camrelizumab for unresectable hepatocellular carcinoma is superior to early combination. BMC Cancer 22(1): 335. <https://doi.org/10.1186/s12885-022-09451-1>

S14. Xin Y, Cao F, Yang H, Zhang X, Chen Y, Cao X et al (2022) Efficacy and safety of atezolizumab plus bevacizumab combined with hepatic arterial infusion chemotherapy for advanced hepatocellular carcinoma. Front Immunol 13: 929141. <https://doi.org/10.3389/fimmu.2022.929141>

S15. Zhu AX, Finn RS, Edeline J, Cattan S, Ogasawara S, Palmer D et al (2018) Pembrolizumab in patients with advanced hepatocellular carcinoma previously treated with sorafenib (KEYNOTE-224): a non-randomised, open-label phase 2 trial. Lancet Oncol 19(7): 940-952. <https://doi.org/10.1016/s1470-2045(18)30351-6>

S16. Verset G, Borbath I, Karwal M, Verslype C, Van Vlierberghe H, Kardosh A et al (2022) Pembrolizumab Monotherapy for Previously Untreated Advanced Hepatocellular Carcinoma: Data from the Open-Label, Phase II KEYNOTE-224 Trial. Clin Cancer Res 28(12): 2547-2554. <https://doi.org/10.1158/1078-0432.Ccr-21-3807>

S17. Tomonari T, Tani J, Sato Y, Tanaka H, Tanaka T, Taniguchi T et al (2023) Initial therapeutic results of atezolizumab plus bevacizumab for unresectable advanced hepatocellular carcinoma and the importance of hepatic functional reserve. Cancer Med 12(3): 2646-2657. <https://doi.org/10.1002/cam4.5145>

S18. Tada T, Kumada T, Hiraoka A, Kariyama K, Tani J, Hirooka M et al (2023) New prognostic system based on inflammation and liver function predicts prognosis in patients with advanced unresectable hepatocellular carcinoma treated with atezolizumab plus bevacizumab: A validation study. Cancer Med 12(6): 6980-6993. <https://doi.org/10.1002/cam4.5495>

S19. Ferris RL, Spanos WC, Leidner R, Gonçalves A, Martens UM, Kyi C et al (2021) Neoadjuvant nivolumab for patients with resectable HPV-positive and HPV-negative squamous cell carcinomas of the head and neck in the CheckMate 358 trial. J Immunother Cancer 9(6): e002568. <https://doi.org/10.1136/jitc-2021-002568>

S20 Powell SF, Gold KA, Gitau MM, Sumey CJ, Lohr MM, McGraw SC et al (2020) Safety and Efficacy of Pembrolizumab With Chemoradiotherapy in Locally Advanced Head and Neck Squamous Cell Carcinoma: A Phase IB Study. J Clin Oncol 38(21): 2427-2437. <https://doi.org/10.1200/jco.19.03156>

S21. Bauml J, Seiwert TY, Pfister DG, Worden F, Liu SV, Gilbert J et al (2017) Pembrolizumab for Platinum- and Cetuximab-Refractory Head and Neck Cancer: Results From a Single-Arm, Phase II Study. J Clin Oncol 35(14): 1542-1549. <https://doi.org/10.1200/jco.2016.70.1524>

S22. Chow LQM, Haddad R, Gupta S, Mahipal A, Mehra R, Tahara M et al (2016) Antitumor Activity of Pembrolizumab in Biomarker-Unselected Patients With Recurrent and/or Metastatic Head and Neck Squamous Cell Carcinoma: Results From the Phase Ib KEYNOTE-012 Expansion Cohort. J Clin Oncol 34(32): 3838-3845. <https://doi.org/10.1200/jco.2016.68.1478>

S23. Black CM, Hanna GJ, Wang L, Ramakrishnan K, Goto D, Turzhitsky V et al (2023) Real-world treatment patterns and outcomes among individuals receiving first-line pembrolizumab therapy for recurrent/metastatic head and neck squamous cell carcinoma. Front Oncol 13: 1160144. <https://doi.org/10.3389/fonc.2023.1160144>

S24. Zandberg DP, Algazi AP, Jimeno A, Good JS, Fayette J, Bouganim N et al (2019) Durvalumab for recurrent or metastatic head and neck squamous cell carcinoma: Results from a single-arm, phase II study in patients with ≥25% tumour cell PD-L1 expression who have progressed on platinum-based chemotherapy. Eur J Cancer 107: 142-152. <https://doi.org/10.1016/j.ejca.2018.11.015>

S25. Kim H, Kwon M, Kim B, Jung HA, Sun JM, Lee SH et al (2020) Clinical outcomes of immune checkpoint inhibitors for patients with recurrent or metastatic head and neck cancer: real-world data in Korea. BMC Cancer 20(1): 727. <https://doi.org/10.1186/s12885-020-07214-4>

S26. Leddon JL, Gulati S, Haque S, Allen C, Palackdharry S, Mathews M et al (2022) Phase II Trial of Adjuvant Nivolumab Following Salvage Resection in Patients with Recurrent Squamous Cell Carcinoma of the Head and Neck. Clin Cancer Res 28(16): 3464-3472. <https://doi.org/10.1158/1078-0432.Ccr-21-4554>

S27. Seiwert TY, Burtness B, Mehra R, Weiss J, Berger R, Eder JP et al (2016) Safety and clinical activity of pembrolizumab for treatment of recurrent or metastatic squamous cell carcinoma of the head and neck (KEYNOTE-012): an open-label, multicentre, phase 1b trial. Lancet Oncol 17(7): 956-965. <https://doi.org/10.1016/s1470-2045(16)30066-3>

S28. Colevas AD, Bahleda R, Braiteh F, Balmanoukian A, Brana I, Chau NG et al (2018) Safety and clinical activity of atezolizumab in head and neck cancer: results from a phase I trial. Ann Oncol 29(11): 2247-2253. <https://doi.org/10.1093/annonc/mdy411>

S29. Ferris RL, Blumenschein G, Jr., Fayette J, Guigay J, Colevas AD, Licitra L et al (2018) Nivolumab vs investigator's choice in recurrent or metastatic squamous cell carcinoma of the head and neck: 2-year long-term survival update of CheckMate 141 with analyses by tumor PD-L1 expression. Oral Oncol 81: 45-51. <https://doi.org/10.1016/j.oraloncology.2018.04.008>
